# AKT3 drives adenoid cystic carcinoma development in salivary glands

**DOI:** 10.1002/cam4.1293

**Published:** 2017-12-28

**Authors:** Katalin Zboray, Julian Mohrherr, Patricia Stiedl, Klemens Pranz, Laura Wandruszka, Beatrice Grabner, Robert Eferl, Richard Moriggl, Dagmar Stoiber, Kazuhito Sakamoto, Kay‐Uwe Wagner, Helmut Popper, Emilio Casanova, Herwig P. Moll

**Affiliations:** ^1^ Ludwig Boltzmann Institute for Cancer Research (LBI‐CR) Vienna Austria; ^2^ Institute of Cancer Research Medical University of Vienna Comprehensive Cancer Center (CCC) Vienna Austria; ^3^ Institute of Animal Breeding and Genetics University of Veterinary Medicine Vienna Austria; ^4^ Medical University of Vienna Vienna Austria; ^5^ Institute of Pharmacology Center for Physiology and Pharmacology Medical University of Vienna Vienna Austria; ^6^ Eppley Institute for Research in Cancer and Allied Diseases University of Nebraska Medical Center Omaha Nebraska; ^7^ Institute of Pathology Research Unit Molecular Lung and Pleura Pathology Medical University of Graz Graz 8036 Austria; ^8^ Department of Physiology Center of Physiology and Pharmacology Comprehensive Cancer Center Medical University of Vienna Vienna Austria

**Keywords:** Adenoid cystic carcinoma, AKT3, MMTV‐tTA mouse model, oncogene addiction, salivary gland cancer

## Abstract

Salivary gland cancer is an aggressive and painful cancer, but a rare tumor type accounting for only ~0.5% of cancer cases. Tumors of the salivary gland exhibit heterogeneous histologic and genetic features and they are subdivided into different subtypes, with adenoid cystic carcinomas (ACC) being one of the most abundant. Treatment of ACC patients is afflicted by high recurrence rates, the high potential of the tumors to metastasize, as well as the poor response of ACC to chemotherapy. A prerequisite for the development of targeted therapies is insightful genetic information for driver core cancer pathways. Here, we developed a transgenic mouse model toward establishment of a preclinical model. There is currently no available mouse model for adenoid cystic carcinomas as a rare disease entity to serve as a test system to block salivary gland tumors with targeted therapy. Based on tumor genomic data of ACC patients, a key role for the activation of the PI3K‐AKT‐mTOR pathway was suggested in tumors of secretory glands. Therefore, we investigated the role of *Akt3* expression in tumorigenesis and report that *Akt3* overexpression results in ACC of salivary glands with 100% penetrance, while abrogation of transgenic *Akt3* expression could revert the phenotype. In summary, our findings validate a novel mouse model to study ACC and highlight the druggable potential of AKT3 in the treatment of salivary gland patients.

## Introduction

The serine/threonine kinase AKT is a critical effector downstream of the phosphoinositide 3‐kinase (PI3K)‐AKT‐mTOR pathway, and it is also a vulnerable node to be targeted once hyperactivated in tumorigenesis. Its expression and activation controls cellular processes such as cell growth, proliferation, cell survival, and neo‐vascularization and was shown to mediate cancer progression 1, 2. The AKT family consists of three different but highly homologous gene products AKT1, AKT2, and AKT3, which are considered to be attractive targets for the design of small molecule‐based anticancer therapies 3, 4. However, more recent studies demonstrate isotype‐specific, opposing functions of individual isoforms in cancer 5, 6, 7, 8, 9, 10, and a better understanding of isoform‐specific functions is a prerequisite for AKT targeting therapies. Being maybe the least studied AKT isoform, AKT3 was found to be upregulated in estrogen receptor‐deficient breast cancer and androgen‐insensitive prostate cancer cell lines 11, 12. Moreover, knockdown of AKT isoforms has been reported to abrogate invasive growth of salivary gland cancer (SGC) cell lines 13.

Salivary gland cancers are a rare group of malignancies accounting for <0.5% of all cancers and around 3‐5% of all head and neck cancers. The World Health Organization (WHO) distinguishes between 24 subtypes of SGC, and all of them exhibit different morphological and pathological features. SGC predominantly arise in one of the three major salivary glands (submandibular, sublingual, and parotid gland), with adenoid cystic carcinomas (ACC), mucoepidermoid carcinomas, and polymorphous low‐grade adenocarcinomas being the most abundant SGC subtypes 14, 15. Generally, salivary gland tumors are surgically resected. However, nonresectable, recurrent, and metastatic high‐grade SGC respond only weakly to cytotoxic chemotherapy, and targeted therapies are not available in most SGC subtypes, culminating in poor prognosis 16, 17, 18. In order to develop targeted therapies in this field, a better understanding of salivary gland tumorigenesis is required, and more recent studies addressing the mutational landscape of SGC may facilitate the identification of novel oncogenic drivers in SGC 19, 20. Intriguingly, these studies revealed mutations in genes implicated in the PI3K‐AKT‐mTOR signaling cascade, suggesting a prominent role of this pathway in salivary gland tumorigenesis. Indeed, recurrent mutations resulting in activation of the PI3K‐AKT‐mTOR pathway were found in 30% of ACC^21^, and activated (phosphorylated) AKT isoforms and downstream mTOR is enhanced in ACC as compared to healthy adjacent tissue 22, 23.

Being a central signal mediator in the PI3K‐AKT‐mTOR pathway, we aimed to investigate a possible implication of AKT3 signaling in breast and salivary gland tumorigenesis. Hence, we generated transgenic mice conditionally expressing *Akt3* under the control of the mouse mammary tumor virus long terminal repeat (*MMTV‐LTR*) promoter, which directs expression to the mammary and salivary glands 24, 25.

## Material and Methods

### Animals

For the establishment of TetO‐*Akt3* transgenic mice, a cDNA encoding the murine *Akt3* containing an N‐terminal myristoylation signal and a C‐terminal HA tag was cloned in an expression vector containing the tetracycline‐responsive Tet‐op promoter and an IRES‐luciferase 25. Plasmid DNA was linearized and microinjected into the pronucleus of FVB/N oocytes. TetO‐Akt3 founders were identified by PCR and a TetO‐*Akt3* transgenic line was established in the FVB/N genetic background. For the generation of MMTV‐tTA/TetO‐*Akt3* double transgenic animals TetO‐*Akt3* transgenic were crossed with MMTV‐tTA mice 25. In all described experiments, littermates were used as controls. All mice were kept and bred under standardized conditions according to an ethical animal license protocol complying with the current Austrian Law. Genotyping of the mice was performed using the primer pairs (P1/P2) for detection of the Akt3‐transgene and the primer pairs (P3/P4) for detection of the MMTV promoter (see Table S1).

### RNA and real‐time quantitative PCR

RNA was isolated with TRIzol Reagent (Life Technologies, Rockford, IL, USA) according to the manufacturer's instructions. RNA was treated with DNaseI (Thermo Fisher Scientific, Waltham, MA, USA) prior to reverse transcription by RevertAid First Strand cDNA Synthesis Kit (Thermo Fisher Scientific) using random hexamer primers. cDNA derived from 50 ng total RNA was used per reaction. qRT‐PCR was performed using SYBR Green. Mouse *Actb* and 28S as housekeeping controls were detected using the primer pairs P5/P6 and P7/P8, respectively. *Akt3* transgene detection was performed using the primer pairs P9/P10 (see Table S1).

### Western blot analysis

Salivary gland tissue homogenates were prepared from snap‐frozen salivary gland tissues in RIPA buffer (Cell signaling technology, Danvers, MA, USA Cat.No‐ 9806). Tissue homogenates were cleared by centrifugation at 4°C for 15 min at 15.0000*g*. Protein concentration was determined using the Bradford protein assay method (Bio‐Rad, Hercules, CA, USA) Protein Assay Kit I Cat.No‐5000001). Fifty microgram of protein was resolved by SDS‐page and transferred to PVDF‐membranes (GE Healthcare, Chicago, IL, USA). Membranes were blocked with 5% BSA in Tris‐Buffered saline, 0.1% Tween 20. for 1 h followed by primary antibody incubation at 4°C overnight. HA‐Tagged AKT3 was detected using anti‐HA‐Tag primary antibody (1:1000, Rabbit mAb, Cell signaling technology, Cat‐No‐ 3724). Incubated membranes were washed with TBS‐T and probed with Anti‐rabbit IgG, HRP‐linked secondary antibody (1:5000, Cell signaling technology, Cat‐No‐7074). Signal detection was performed using Super Signal West Femto and Pico Kits (Life Technologies). As loading control, anti‐ *β*‐Actin primary antibody was used (1:1000, Rabbit mAb, Cell signaling technology, Cat‐No‐4970).

### Histology

Mouse salivary gland tissues were fixed in 4% formaldehyde solution and embedded into paraffin. Five‐micrometer thick tissue sections were deparaffinized and rehydrated. Sections were either stained with hematoxylin and eosin or subjected to immunohistochemical staining. Stained slides were scanned with TissueFaxs software (TissueGnostics Gmbh, Vienna, Austria). Quantification of the tumor area, tumor burden, and staining intensities was done with HistoQuest software (TissueGnostics Gmbh). For immunohistochemical stainings, antigen retrieval was performed using Target Retrieval Solution, pH 6.0 (Dako, Santa Clara, CA, USA). Endogenous peroxidase activity was diminished by incubating sections with 3% hydrogen peroxide for 10 min. Sections were blocked with M.O.M blocking solution (Vector Laboratories, Burlingame, CA, USA) for 1 h prior to primary antibody incubation. Used primary antibodies were anti‐HA Tag (1:1000, Rabbit mAb, Cell signaling technology, Cat‐No‐2367), anti‐Ki‐67 (1:400, Rabbit mAb, Cell signaling technology, Cat‐No‐9027), anti‐*α*‐SMA (1:200, Mouse mAb, MS‐113‐P0), and anti‐CC3 (1:300, Rabbit Ab, Cell signaling technology, Cat‐No‐9661). Signal detection was performed using IDetect^™^ Universal Mouse Kit‐HRP (Empire genomics) and 3,3′‐Diaminobenzidin (DAB) as chromogenic substrate.

### Dox treatment of mice

Dt mice with established salivary gland tumors were treated with Dox (Sigma Aldrich, Cat‐No‐D9891**)** for three consecutive weeks. Dox was applied at a final concentration of 1 mg/mL Dox in drinking water containing 1% sucrose (Sigma Aldrich, Cat‐No‐ S0389). The prepared solution was changed twice a week. Measurement of the tumor volume was performed every second day using a caliper. Tumor volume was calculated using the following equation: (width*width*length)/2.

### Human data

We used the cBioPortal for Cancer Genomics browser 26, 27 to analyze the TCGA dataset for head and neck cancer patients 28. To identify patients with increased AKT3 mRNA expression, we applied a z‐score of ±1.5 RNASeq V2 RSEM. We also used the publically available GEO dataset GSE10300 for analysis. Probe set for *AKT3* was 212607_at.

### Statistics

All values are given as means ± SD. Comparison between two groups was made by Student`s *t*‐test. For Kaplan–Meier analysis, a log‐rank test was performed.

## Results

### Akt3 overexpression triggers salivary gland tumor formation

**Figure 1:**
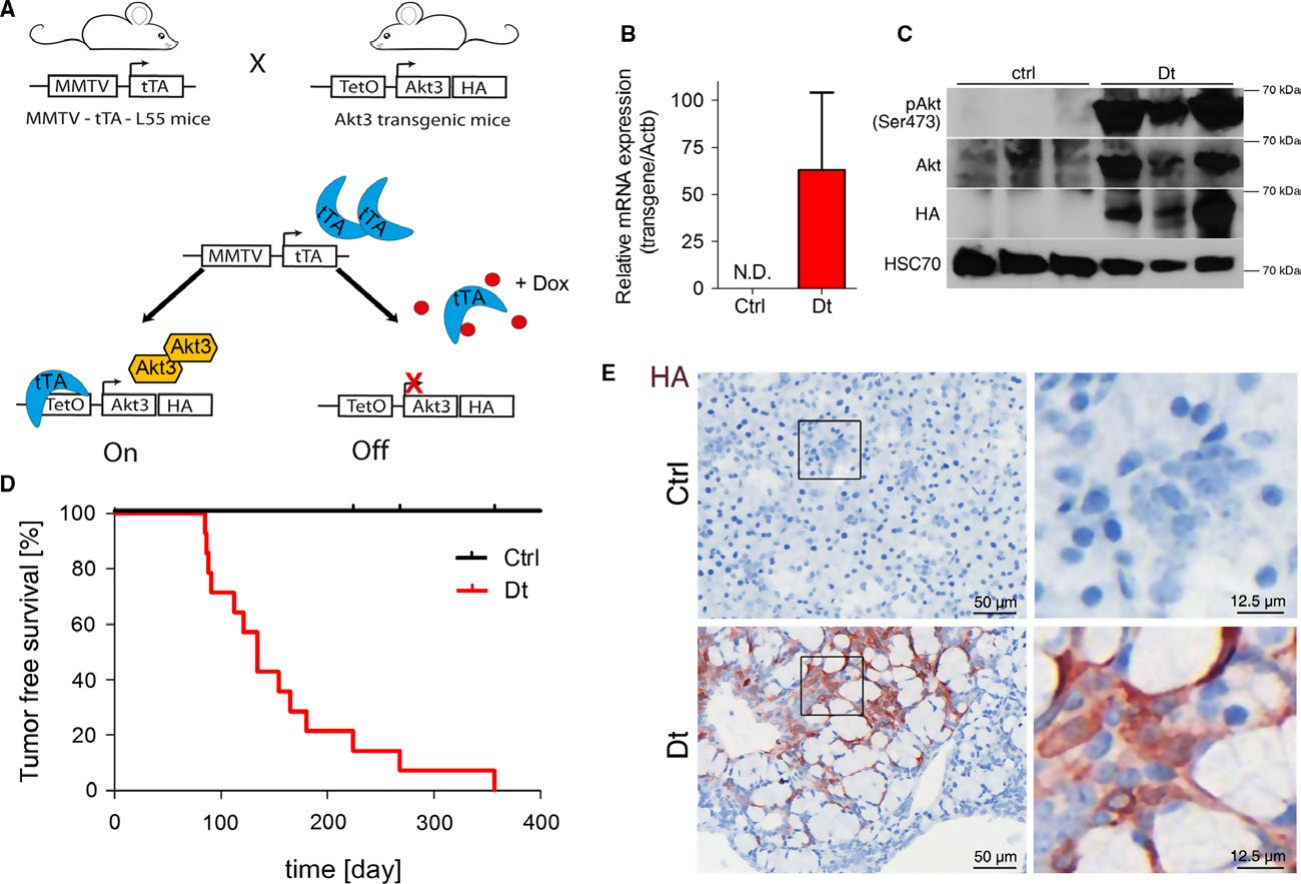
Akt3 overexpression provokes salivary gland tumorigenesis: (A) Scheme depicting breeding strategy of MMTV‐driven expression of HA‐tagged Akt3 via tTA. Dox administration represses transgenic Akt3 expression. (B) Expression of the Akt3 transgene in salivary gland tissues of Dt animals was verified by qRT‐PCR (*n* = 5) analysis and (C) by western blot analysis. (D) Kaplan–Meier plot depicting time until tumors were clearly visible in Ctrl and Dt mice (*n* > 10 mice per group). (E) IHC of salivary glands of Ctrl and Dt mice probed for HA expression. Pictures on the right show a higher magnification of the same sections.

We generated *TetO‐Akt3* transgenic mice by injecting linearized plasmid DNA encoding the mouse *Akt3* cDNA containing an N‐terminal myristoylation signal and a C‐terminal Human influenza hemagglutinin (HA)‐tag under control of the tetracycline‐responsive Tet‐op promoter and an *IRES‐luciferase* into the pronucleus of FVB/N oocytes. To study the role of *Akt3* in salivary and mammary gland tumorigenesis, we then took advantage of the Tet‐Off system and crossed *MMTV‐tTA* transgenic mice 25 with *TetO‐Akt3* transgenic mice. Breeding these strains lead to the generation of *MMTV‐tTA/TetO‐Akt3* double transgenic animals (hereafter: Dt). These mice show sustained expression of HA‐tagged Akt3 in mammary and salivary glands which can be switched off by Doxycycline (Dox) treatment (Fig. 1A).

To validate the model, we performed real‐time quantitative PCR analysis (qRT‐PCR) using primers specific for the transgene and confirmed transgenic mRNA expression in the Dt mouse group at 8 weeks of age (Fig. 1B). On the protein level, we confirmed expression of the HA‐tagged Akt3 which resulted into increased levels of total Akt protein, verified by an antibody against pan‐Akt. Notably, we detected massive activation of Akt in Dt mice, which was absent in wild‐type ctrl mice (Fig. 1C). Intriguingly, Dt mice suffered from the formation of salivary gland cancer with 100% penetrance and with a median tumor‐free survival of 134 days (Fig. 1D). Tumors isolated from Dt mice stained positive for HA‐tagged Akt3 by immunohistochemistry (IHC) (Fig. 1E). At the time of sacrifice due to tumor burden and according to the animal law, mammary glands of Dt mice did not exhibit any malignant phenotype and Ctrl mice were tumor‐free (data not shown). These data suggest an essential function of *Akt3* in the rapid and aggressive development of salivary gland tumors. Hence, these results did not only validate the transgenic mouse model used in this study, but also confirmed an oncogenic role for high *Akt3* expression as driver for salivary gland tumor progression.

### *Akt3*‐driven salivary gland tumors exhibit adenoid cystic carcinoma characteristics

**Figure 2:**
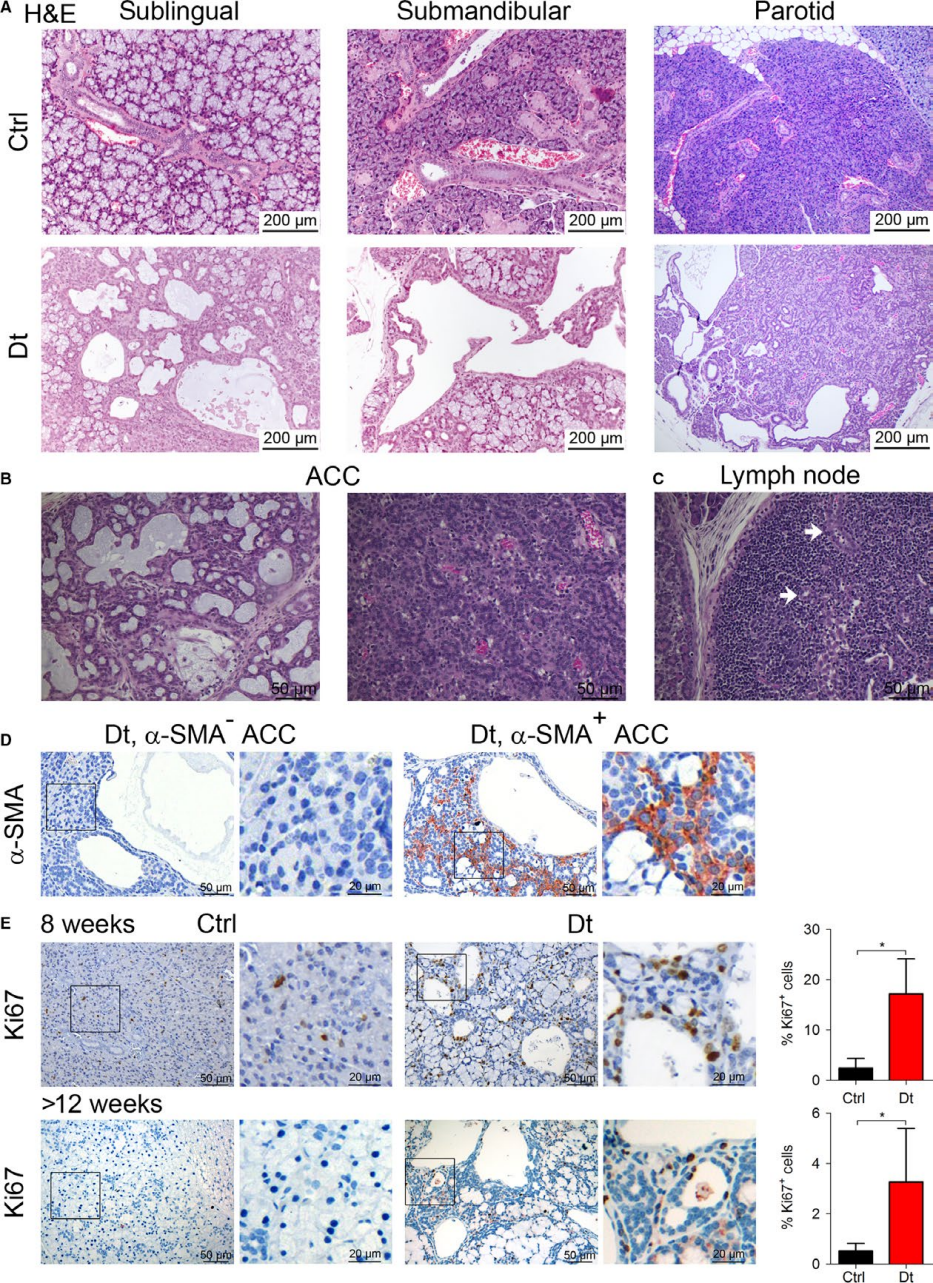
Tumors show an adenoid cystic carcinoma pathology. (A) Representative H&E stainings of sublingual, submandibular, and parotid glands in Ctrl versus Dt mice. (B). H&E staining of adenoic cystic carcinoma of the salivary gland depicting the typical large cystic spaces and small pseudoglandular spaces covered by cuboidal cells (left) and microglandular pattern with solid areas. The proteinaceous material is stained in violet. (C) H&E staining of a newly formed lymph node. White arrows indicate infiltrating carcinomas. (D) ACC in Dt mice show negative and positive areas for *α*‐SMA staining (E) Representative pictures of immunohistochemistry for the proliferation marker Ki67 of salivary gland sections of Ctrl and tumor‐bearing Dt mice at 8 weeks and >12 weeks of age. Ki67^+^ were quantified using TissueQnostic software. Data are presented as mean ± sd and were analyzed by Student's *t*‐test (*n* = 3, *P < 0.05).

To characterize the tumors, we dissected the major salivary glands and consecutive hematoxylin‐eosin (H&E)‐stained tissue sections were examined by a board‐certified pathologist (HP). All tumors analyzed displayed similar immunopathologic features and were classified as adenoid cystic carcinomas (ACC). ACC were found in all major salivary glands, that is, the sublingual, the submandibular, and parotid glands, and ACC formation led to complete disruption of the normal histological architecture of the salivary gland (Fig. 2A). Indeed, the histopathology of the glands of Dt mice indicates nodular proliferation of uniform basaloid cells, consistent with ACC. Typical large cystic spaces can be seen including pseudoglandular spaces covered by cuboidal cells as well as areas with microglandular patterns (Fig. 2B). Furthermore, in the pseudolumina, a mucus‐like material is present, which is composed of proteinaceous fluid, containing mainly fibronectin and collagen type IV (Fig. 2B). Infiltration of the carcinoma into a newly formed lymph node confirms the malignant potential of this tumor (Fig. 2C). However, perineural invasion and metastasis, for example, in the lung, as often observed in human patients suffering from ACC 29, 30, 31, were not observed when we sacrificed tumor‐bearing mice. Moreover, we found tumor areas positive for *α*‐smooth muscle actin (*α*‐SMA), a feature of ACC pathology (Fig. 2D) 14. Next, we checked proliferation in salivary gland tissue of Dt mice compared to wild‐type Crtl mice by IHC staining for Ki‐67. We detected significant higher proliferation rate in tumor‐bearing glands in Dt mice, both in 8 week‐old mice and in mice older than 12 weeks, further confirming the contribution of *Akt3* to ACC progression (Fig. 2E). Notably, we did not notice tumor formation in the minor salivary gland, presumably because of insufficient transgene expression.

### Abrogation of Akt3 overexpression mediates tumor regression

**Figure 3:**
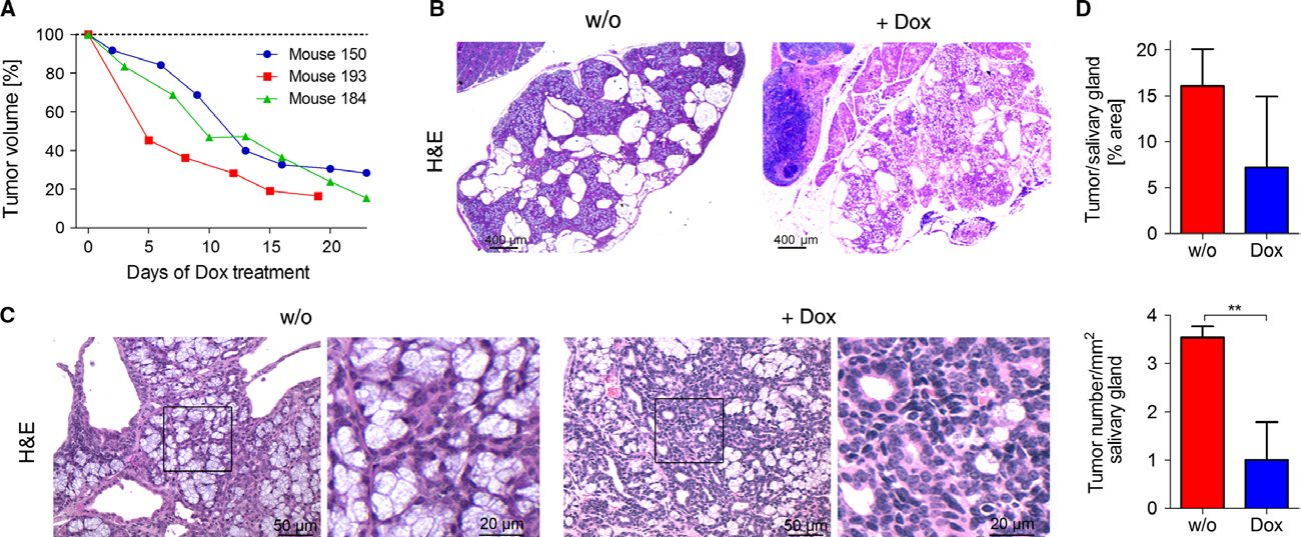
Dox treatment leads to regression of established tumors. (A). Relative tumor volume upon doxycycline (Dox) treatment of mice measured using a caliper. Treatment was started when tumors reached a volume of 500–750 mm^2^ (B & C). Representative H&E staining of ACCs in salivary glands of Dt mice w/o treatment and with Dox treatment. (D) Quantitation of tumor area and tumor number using TissueQnostic software. Graphs represent mean ± SD, data were analyzed by Student's *t*‐test. (*n* = 3, **P < 0.01).

**Figure 4:**
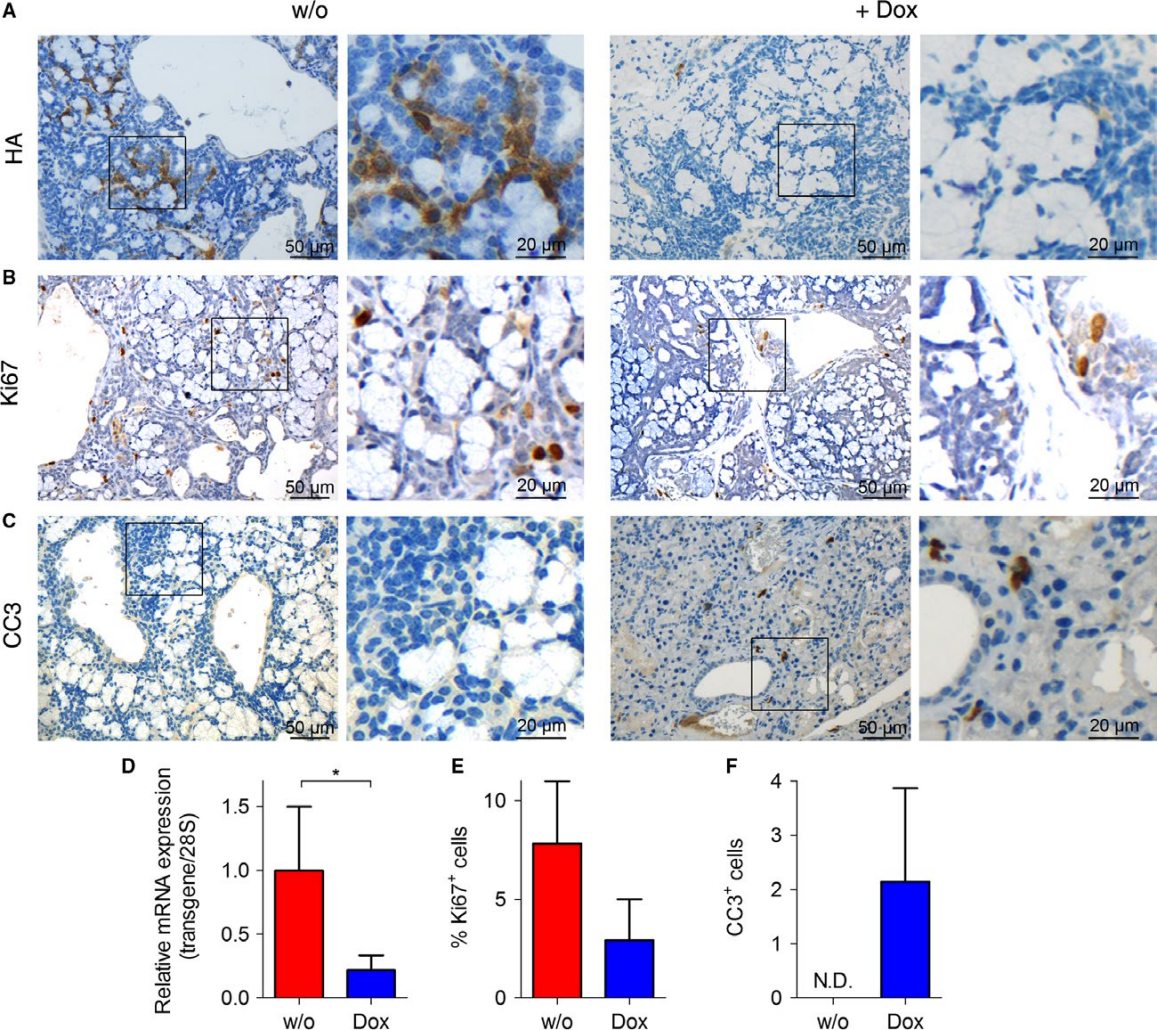
Abrogation of Akt3 overexpression reduces proliferation and triggers apoptosis: Representative pictures of immunohistochemistry for expression of (A) The HA‐tagged Akt3 transgene, (B) Ki67, and (C) cleaved Caspase 3 (CC3). (D) Downregulation of the Akt3 transgene in salivary gland tissues upon Dox treatment of Dt animals was verified by qRT‐PCR (*n* = 4 ctrl and 3 dox). (E) Quantitation of Ki67 and (F) CC3 immunohistochemistry was analyzed using TissueQnostic software. Graphs represent mean ± SD, data were analyzed by Student's *t*‐test. (*n* = 3, *P < 0.05).

Next, we tested whether rising ACC become addictive to *Akt3* overexpression by taking advantage of our Dt mouse model. Hence, we treated salivary gland tumor‐harboring mice with doxycycline (Dox), thereby abrogating transgenic *Akt3* overexpression. Administration of Dox immediately triggered regression of tumors in all mice tested (*n* = 3, Fig. 3A). Indeed, we observed reduction in tumor burden and tumor number by analysis of H&E‐stained salivary gland sections and normalization of the salivary gland structure following Dox treatment (Fig. 3B–D). Regression of tumors correlated with decreased expression of the Akt3 transgene in the salivary glands as verified by IHC staining of the HA tag and by qPCR (Fig. 4A and D) and was not only reflected by decreased proliferation of tumors in Dox‐treated mice as tested by Ki‐67 staining (Fig. 4B and E), but also by increased apoptosis. Abrogation of *Akt3* overexpression triggered activation of the apoptotic cascade, as evidenced by positive IHC staining for cleaved caspase 3, which was completely absent in non‐Dox‐treated Dt mice (Fig. 4C and E). Altogether, these data demonstrate the dependence of ACC tumorigenesis on *Akt3* expression and further validates our mouse model as a potent tool to study *Akt3*‐driven salivary gland tumorigenesis.

## Discussion

The *MMTV* promoter has been widely used to conditionally manipulate gene expression in secretory glands and serves as a valuable tool to study disease in salivary and mammary glands 25, 32, 33, 34, 35. We took advantage of this model by crossing the *MMTV‐tTA* mouse 25 into mice expressing *Akt3* under *TetO* control, which were generated in our laboratory for this study. Dt mice developed salivary gland ACC within a few weeks of age, which became clearly visible within 4–5 months.

The clinical management of SGC remains still challenging. Major obstacles for a better outcome in the treatment of SGC patients are high recurrence rates and the high metastatic potential of these tumors, as well as their poor response to chemotherapy 36. Recent exon and whole genome sequences suggest alternative treatment strategies targeting abundant *MYB‐NFIB* fusion oncogenes, or genes involved in NOTCH1 or FGF receptor signaling, as well as targeting the PI3K/AKT/mTOR pathway^21^, ^37^, ^38^. However, drugs for targeted therapies have not overcome the hurdles into clinical application for SGC yet. Indeed, seeking for novel treatment options using small molecule inhibitors is difficult for rare diseases such as SGC in general and salivary ACC in particular, since adequate preclinical models are barely available. This work demonstrates the pro‐oncogenic potential of *Akt3* in salivary glands to drive ACC in a novel genetically modified mouse model. These data are in line with a previous report showing that siRNA‐mediated *AKT3* knockdown limits invasive growth of human salivary gland cell lines 13. Also, highly phosphorylated AKT levels in ACC tissue were associated with an increased risk for tumor relapse 23. In contrast, a recent publication showed that activation of AKT was also associated with better prognosis of salivary gland ACC patients 22. This discrepancy might arise because of distinct functions of the different AKT isoforms, suggesting the need for AKT isoform‐specific inhibitors in the treatment of salivary gland ACC patients 39.

Indeed, despite the high sequence similarity of the family members, different functions for the AKT isoforms were reported with respect to cancer. For example, AKT1 was revealed as an oncogene in mammary cancer, and AKT2 primarily acts on metastatic dissemination^5^, ^40^, ^41^ In contrast, AKT3, but not AKT1 and AKT2, was required for the growth of triple‐negative breast cancer cell lines 12. However, the genetic landscape in breast ACC compared to salivary gland ACC is different, and so are morphologic and clinical features 42, 43. In our model, the dominant phenotype in salivary glands precluded us to investigate the role of *Akt3* in mammary gland tumorigenesis. We acknowledge that it is unclear whether our model assures adequate *Akt3* expression in mammary glands during the whole life span of our mice, and whether expression levels achieved would be sufficient to drive mammary gland tumorigenesis. Therefore, we want to emphasize that our work by no means rules out an oncogenic role of *Akt3* in development of cancers of the mammary gland.

Altogether, our data demonstrate the potent oncogenic role of *AKT3* for ACC pathogenesis in vivo. Furthermore, our novel mouse model has the potential to serve as a valuable tool to study salivary gland ACC and develop new therapeutic strategies.

## Conflict of Interest

The authors declare no potential conflict of interest.

## Supporting information


**Table S1**. Sequences of primers used for genotyping and SYBR green‐based qRT‐PCR.
