# Supplementary material for: AKT3 drives adenoid cystic carcinoma development in salivary glands
Source: Cancer Med. 2017 Dec 28;7(2):445–53. doi: 10.1002/cam4.1293 (PMC5806106; doi:10.1002/cam4.1293)
Supplement: Supplementary file 1 — Table S1. Sequences of primers used for genotyping and SYBR green‐based qRT‐PCR. [file CAM4-7-445-s001.docx]

**sTables**

Table 1: Sequences of primers used for genotyping and SYBR green based qRT-PCR

| Primer | 5´-3´sequence |
| --- | --- |
| P1 | GTTCGTCACATCTCATCTACCTCCCGG |
| P2 | CGACTCGAAATCCACATATCAAATATCC |
| P3 | CGCTAGACGATTTCGATCTGG |
| P4 | CTCCCATTCATCAGTTCCATAGG |
| P5 | GTTAATGGCGGAGAGCTGTT |
| P6 | TACTCTGGTGTGCCACAGAA |
| P7 | ATACCGGCACGAGACCGATAGTCA |
| P8 | GCGGACCCCACCCGTTTACCTC |
| P9 | GCTCATAGCTCTTCTCCAGGG |
| P10 | CCTGAACCCTAAGGCCAACCG |
